# Supplementary material for: Genome-Wide Analysis of the Growth-Regulating Factor (GRF) Family in Aquatic Plants and Their Roles in the ABA-Induced Turion Formation of Spirodela polyrhiza
Source: Int J Mol Sci. 2022 Sep 10;23(18):10485. doi: 10.3390/ijms231810485 (PMC9499638; doi:10.3390/ijms231810485)
Supplement: Supplementary file 1 [file ijms-23-10485-s001.zip › ijms-1905434-supplementary.pdf]

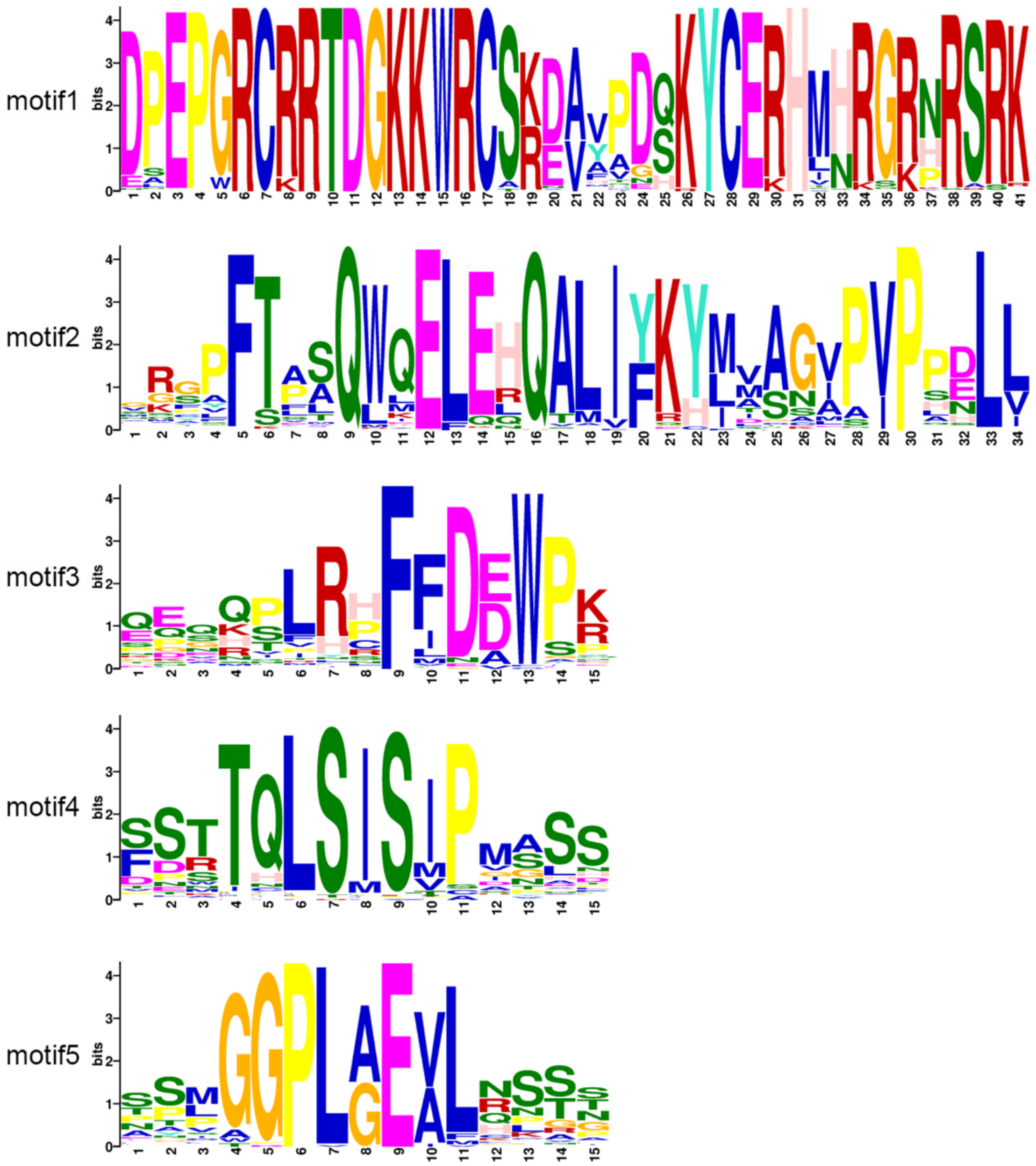

Figure S1. Characteristics of five putative conserved motifs in 139 GRFs.

Table S1. Gene information, predicted putative physiochemical properties, NLSs and subcellular localizations of 139 GRF proteins.

Gene name	Gene ID	Species	Protein length (aa)	MW(KDa)	PI	Predicted subcellular localization	Predicted monopartite NLS (position)	Predicted bipartite NLS (position)
AfGRF1	Azfi_s0001.g000094	*Azolla filiculoides*	625	67.1502	6.61	Nucleus	-	273
AfGRF2	Azfi_s0097.g043820	*Azolla filiculoides*	801	82.10981	6.66	Nucleus	-	239
AfGRF3	Azfi_s0256.g060519	*Azolla filiculoides*	818	87.30616	6.72	Nucleus	-	372
AtGRF1	AT2G22840	*Arabidopsis thaliana*	530	56.40134	9.27	Nucleus	-	201; 325; 348
AtGRF2	AT4G37740	*Arabidopsis thaliana*	535	58.58489	8.85	Nucleus	-	232; 256; 440
AtGRF3	AT2G36400	*Arabidopsis thaliana*	398	43.70729	8.56	Nucleus	-	4; 149
AtGRF4	AT3G52910	*Arabidopsis thaliana*	380	42.53372	6.87	Nucleus	-	156; 250
AtGRF5	AT3G13960	*Arabidopsis thaliana*	397	44.69557	8.18	Nucleus	-	86
AtGRF6	AT2G06200	*Arabidopsis thaliana*	244	28.21061	8.87	Nucleus	-	85; 196
AtGRF7	AT5G53660	*Arabidopsis thaliana*	365	40.41032	8.13	Nucleus	-	121; 140
AtGRF8	AT4G24150	*Arabidopsis thaliana*	493	54.6087	6.44	Nucleus	-	-
AtGR	AT2G45480	*Arabido*	429	48.60	8.3	Nucleu	-	92;

F9		*psis thaliana*		757	5	s		310
AvGR F1	Av_00000935-RA	*Aldrovanda vesiculosa*	369	41.65 316	8.7 7	Nucleus	-	84; 263
AvGR F10	Av_00011365-RA	*Aldrovanda vesiculosa*	367	41.17 092	8.7 2	Nucleus	-	84; 263 265
AvGR F11	Av_00013002-RA	*Aldrovanda vesiculosa*	374	41.99 66	9.1 1	Nucleus	-	83
AvGR F12	Av_00013064-RA	*Aldrovanda vesiculosa*	455	49.98 494	8.5 5	Nucleus	-	106; 115
AvGR F13	Av_00014513-RA	*Aldrovanda vesiculosa*	503	53.02 958	8.5 8	Nucleus	-	-
AvGR F14	Av_00015655-RA	*Aldrovanda vesiculosa*	355	38.99 746	7.7	Nucleus	132	106; 130; 130
AvGR F15	Av_00018870-RA	*Aldrovanda vesiculosa*	541	57.59 941	8.9 4	Cell membrane. Nucleus	-	316
AvGR F16	Av_00018901-RA	*Aldrovanda vesiculosa*	409	44.18 537	9.1 1	Nucleus	-	91
AvGR F17	Av_00020000-RA	*Aldrovanda vesiculosa*	565	60.05 906	8.1 1	Cell membrane	-	-
AvGR F18	Av_00020017-RA	*Aldrovanda vesiculosa*	229	25.26 819	9.2 4	Nucleus	-	79
AvGR	Av_00022695-	*Aldrova*	601	63.66	9.1	Cell	-	239;

F19	RA	*nda vesiculosa*		055	7	membrane. Nucleus		263
AvGRF2	Av_00004307-RA	*Aldrovanda vesiculosa*	362	40.49102	8.94	Nucleus	-	85; 259
AvGRF20	Av_00023828-RA	*Aldrovanda vesiculosa*	410	44.9137	6.33	Nucleus	-	179
AvGRF21	Av_00024262-RA	*Aldrovanda vesiculosa*	628	67.20784	8.35	Cell membrane. Nucleus	-	266; 290; 392
AvGRF3	Av_00004572-RA	*Aldrovanda vesiculosa*	406	45.1023	8.92	Nucleus	-	42; 108; 265
AvGRF4	Av_00007247-RA	*Aldrovanda vesiculosa*	372	41.47896	8.37	Nucleus	-	83
AvGRF5	Av_00009217-RA	*Aldrovanda vesiculosa*	560	58.98234	8.18	Cell membrane	-	-
AvGRF6	Av_00009484-RA	*Aldrovanda vesiculosa*	543	57.48387	7.52	Cell membrane	-	197; 302
AvGRF7	Av_00009515-RA	*Aldrovanda vesiculosa*	401	43.76747	8.69	Nucleus	-	113
AvGRF8	Av_00010330-RA	*Aldrovanda vesiculosa*	518	55.06272	7.58	Nucleus	-	-
AvGRF9	Av_00010799-RA	*Aldrovanda vesiculo*	518	55.60684	8.18	Cell membrane.	-	25; 202

		sa				Nucleus		
CcGRF1	GWHPACFD000460	*Cladopus chinensis*	329	35.87144	5.32	Cell membrane. Nucleus	-	119; 128
CcGRF10	GWHPACFD015113	*Cladopus chinensis*	271	30.69875	8.27	Nucleus	-	-
CcGRF11	GWHPACFD022459	*Cladopus chinensis*	263	29.67149	8.65	Nucleus	-	27; 113
CcGRF2	GWHPACFD002369	*Cladopus chinensis*	356	39.23585	8.02	Nucleus	-	170
CcGRF3	GWHPACFD004174	*Cladopus chinensis*	272	30.10859	8.71	Nucleus	-	137; 196
CcGRF4	GWHPACFD005783	*Cladopus chinensis*	246	27.52706	9.53	Nucleus	-	136
CcGRF5	GWHPACFD007337	*Cladopus chinensis*	305	34.40959	8.9	Nucleus	-	80; 89
CcGRF6	GWHPACFD007888	*Cladopus chinensis*	246	27.48202	9.44	Nucleus	-	136
CcGRF7	GWHPACFD012264	*Cladopus chinensis*	382	42.74732	9.35	Nucleus	-	103; 159
CcGRF8	GWHPACFD012674	*Cladopus chinensis*	313	35.47848	7.58	Nucleus	-	201
CcGR	GWHPACFD0	*Cladopu*	270	30.95	6.1	Nucleu	-	77

F9	14476	*s chinensis*		586	9	s		
CdGRF1	prefix00835.t1	*Ceratophyllum demersum*	381	42.58055	8.18	Nucleus	-	150; 159
CdGRF10	prefix15436.t1	*Ceratophyllum demersum*	342	38.4146	8.39	Nucleus	-	76
CdGRF11	prefix17660.t1	*Ceratophyllum demersum*	403	44.98444	5.69	Nucleus	-	134
CdGRF12	prefix18448.t1	*Ceratophyllum demersum*	284	33.45044	8.55	Nucleus	-	79; 88; 238
CdGRF13	prefix21354.t1	*Ceratophyllum demersum*	323	36.36829	7.64	Nucleus	-	61
CdGRF14	prefix25051.t1	*Ceratophyllum demersum*	139	15.85011	9.58	Nucleus	-	50
CdGRF2	prefix01244.t1	*Ceratophyllum demersum*	433	47.8498	6.5	Nucleus	-	16; 144
CdGRF3	prefix04847.t1	*Ceratophyllum demersum*	541	60.72992	6.44	Nucleus	-	251
CdGRF4	prefix05720.t1	*Ceratophyllum demersum*	358	39.42026	6.15	Nucleus	-	-
CdGRF5	prefix06054.t1	*Ceratophyllum demersum*	333	37.59273	7.64	Nucleus	-	76
CdG	prefix06361.t1	*Ceratop*	585	64.47	8.4	Nucleu	-	224

RF6		*hyllum demersum*		395		s		
CdGRF7	prefix07205.t1	*Ceratophyllum demersum*	735	81.64069	9.45	Nucleus	-	223; 580; 650
CdGRF8	prefix09195.t1	*Ceratophyllum demersum*	391	43.26227	6.61	Nucleus	-	81; 105
CdGRF9	prefix14436.t1	*Ceratophyllum demersum*	233	26.23688	9.18	Nucleus	-	129
EfGRF1	g01483.t1	*Euryale ferox*	338	38.00605	8.15	Nucleus	-	78; 87
EfGRF10	g09724.t1	*Euryale ferox*	462	49.75375	9.5	Nucleus	-	31
EfGRF11	g11371.t1	*Euryale ferox*	589	63.26748	8.41	Nucleus	-	33; 220; 457
EfGRF12	g12032.t1	*Euryale ferox*	230	24.68304	9.32	Nucleus	-	88
EfGRF13	g12966.t1	*Euryale ferox*	331	36.9169	8.88	Nucleus	-	72
EfGRF14	g16304.t1	*Euryale ferox*	345	38.36869	9.17	Nucleus	-	83
EfGRF15	g17571.t1	*Euryale ferox*	614	65.23352	8.25	Nucleus	-	200
EfGRF16	g17620.t1	*Euryale ferox*	265	29.07685	9.51	Nucleus	-	137
EfGRF17	g24211.t1	*Euryale ferox*	235	25.31819	9.32	Chloroplast. Nucleus	-	109
EfGRF18	g26131.t1	*Euryale ferox*	310	35.32833	9.05	Nucleus	-	76; 85
EfGRF19	g30061.t1	*Euryale ferox*	587	62.97224	8.5	Nucleus	-	34; 220; 452
EfGRF2	g03460.t2	*Euryale ferox*	307	34.76871	8.9	Nucleus	-	77; 86
EfGR	g36593.t2	*Euryale*	331	36.81	8.5	Nucleu	-	69

F20		*ferox*		882	5	s		
EfGRF3	g03523.t2	*Euryale ferox*	575	61.39942	6.76	Nucleus	-	135; 191
EfGRF4	g03563.t1	*Euryale ferox*	273	29.89865	9.28	Nucleus	-	12; 139
EfGRF5	g04250.t1	*Euryale ferox*	200	21.79967	9.95	Nucleus	-	59; 85
EfGRF6	g07175.t1	*Euryale ferox*	621	65.57756	7.6	Nucleus	-	199
EfGRF7	g07218.t1	*Euryale ferox*	274	30.02184	9.36	Nucleus	-	12; 13; 139
EfGRF8	g08094.t1	*Euryale ferox*	332	36.93589	9.09	Nucleus	-	72
EfGRF9	g08916.t1	*Euryale ferox*	318	34.68476	9.39	Nucleus	-	84
LmGRF1	lm5633.a03.13.g01020_p1	*lemna minuta*	218	23.59446	9.27	Nucleus	-	83
LmGRF2	lm5633.a03.4.g01820_p1	*lemna minuta*	340	38.77158	8.71	Nucleus	-	94; 221; 244
LmGRF3	lm5633.a03.8.g10900_p1	*lemna minuta*	261	28.56218	8.89	Chloroplast. Nucleus	145	103
NcGRF1	XP_031478480.1	*Nymphaea colorata*	589	63.26154	8.6	Nucleus	-	34; 220; 457
NcGRF2	XP_031482096.1	*Nymphaea colorata*	332	36.91385	8.88	Nucleus	-	72
NcGRF3	XP_031482372.1	*Nymphaea colorata*	576	61.4075	8.69	Nucleus	-	219
NcGRF4	XP_031491651.1	*Nymphaea colorata*	338	38.10917	7.7	Nucleus	-	78; 87
NcGRF5	XP_031495439.1	*Nymphaea colorata*	297	31.90194	9.14	Nucleus	-	140
NcGRF6	XP_031498107.1	*Nymphaea colorata*	278	30.23004	9.15	Nucleus	-	140
NcG	XP_031498261	*Nympha*	412	46.98	9.1	Nucleu	-	37;

RF7	.1	*ea colorata*		986	7	s		40; 180; 189
NcGRF8	XP_031499041.1	*Nymphaea colorata*	645	68.47868	8.47	Nucleus	-	215
NcGRF9	XP_031503184.1	*Nymphaea colorata*	333	36.91496	8.92	Nucleus	-	69; 245
NnGRF1	NNU_000550-RA	*Nelumbo nucifera*	581	61.70756	6.36	Cell membrane. Cell wall. Nucleus	-	176
NnGRF2	NNU_001408-RA	*Nelumbo nucifera*	243	25.64872	9.05	Cell membrane. Nucleus	-	101
NnGRF3	NNU_002283-RA	*Nelumbo nucifera*	604	65.565	8.14	Nucleus	-	228; 252
NnGRF4	NNU_002395-RA	*Nelumbo nucifera*	380	41.38598	9.05	Nucleus	-	142
NnGRF5	NNU_006992-RA	*Nelumbo nucifera*	355	39.77916	8.82	Nucleus	-	81
NnGRF6	NNU_010640-RA	*Nelumbo nucifera*	372	42.1876	8.61	Nucleus	-	78; 87; 259; 302
NnGRF7	NNU_014113-RA	*Nelumbo nucifera*	550	58.25181	7.53	Cell membrane. Nucleus	-	205
NnGRF8	NNU_018235-RA	*Nelumbo nucifera*	372	42.10757	8.95	Nucleus	-	79; 255; 282
NnGRF9	NNU_023103-RA	*Nelumbo*	436	47.47062	7.23	Nucleus	-	192

		nucifera						
SiGRF1	CAA7387704.1	*Spirodela intermedia*	530	56.36808	8.27	Nucleus	-	194; 273
SiGRF2	CAA7391974.1	*Spirodela intermedia*	357	38.15435	8.64	Nucleus	-	78; 253
SiGRF3	CAA7393787.1	*Spirodela intermedia*	158	17.22233	10.02	Nucleus	-	68
SiGRF4	CAA7394958.1	*Spirodela intermedia*	300	33.68392	9.21	Nucleus	-	86; 229
SiGRF5	CAA7399847.1	*Spirodela intermedia*	203	21.81505	10.28	Nucleus	-	88; 176
SiGRF6	CAA7401268.1	*Spirodela intermedia*	333	37.09969	8.63	Nucleus	-	85
SiGRF7	CAA7406501.1	*Spirodela intermedia*	372	39.37449	8.48	Nucleus	-	87
SpGRF1	Spo001498	*Spirodela polyrhiza*	525	54.61278	8.64	Nucleus	-	190; 267
SpGRF2	Spo004459	*Spirodela polyrhiza*	301	33.74287	9.22	Nucleus	-	86
SpGRF3	Spo005290	*Spirodela polyrhiza*	559	59.84567	8.74	Nucleus	-	47; 220
SpGRF4	Spo012722	*Spirodela polyrhiz*	402	41.74232	8.79	Nucleus	-	90; 99

		a						
SpGRF5	Spo013751	*Spirodela polyrhiza*	218	22.80673	9.3	Chloroplast. Nucleus	9	111
SpGRF6	Spo015563	*Spirodela polyrhiza*	326	36.35185	8.63	Nucleus	-	86
UgGRF1	unitig_0.g2446.t1	*Utricularia gibba*	266	29.12906	8.86	Nucleus	117	-
UgGRF10	unitig_8.g3578.t1	*Utricularia gibba*	345	39.0202	9.64	Nucleus	-	-
UgGRF11	unitig_8.g4145.t1	*Utricularia gibba*	300	32.92823	8.67	Nucleus	-	90
UgGRF12	unitig_8.g4856.t1	*Utricularia gibba*	454	48.42992	9.06	Nucleus	-	178
UgGRF2	unitig_0.g2836.t1	*Utricularia gibba*	672	73.13494	8.8	Nucleus	301	45; 406
UgGRF3	unitig_21.g20615.t1	*Utricularia gibba*	389	42.59214	9.07	Nucleus	-	65
UgGRF4	unitig_21.g20982.t1	*Utricularia gibba*	347	39.55484	6.99	Nucleus	-	104; 282; 287
UgGRF5	unitig_699.g19428.t1	*Utricularia gibba*	207	23.90758	10.88	Nucleus	-	81; 134
UgGRF6	unitig_744.g24115.t1	*Utricularia gibba*	496	53.4653	7.74	Nucleus	-	169
UgGRF7	unitig_747.g21926.t1	*Utricularia gibba*	751	81.85488	8.7	Nucleus	-	91; 457
UgGRF8	unitig_749.g14242.t1	*Utricularia gibba*	227	25.96334	9.48	Nucleus	-	97
UgGRF9	unitig_749.g14291.t1	*Utricularia*	290	31.89499	7.76	Nucleus	-	95

		gibba						
WaGRF1	Waus00004g13948.1	*Wolffia australiana*	666	73.02658	8.67	Chloroplast	519; 520; 523	248; 257; 504
WaGRF2	Waus00016g05717.1	*Wolffia australiana*	228	26.01245	9.15	Nucleus	-	86
WaGRF3	Waus00022g09139.1	*Wolffia australiana*	478	51.02264	8.86	Cell membrane. Nucleus	-	189
WaGRF4	Waus00029g11302.1	*Wolffia australiana*	206	23.05058	9.53	Nucleus	-	79
WaGRF5	Waus00054g16188.1	*Wolffia australiana*	219	23.95804	7	Nucleus	-	94
ZmGRF1	Zosma1g02640.1	*Zostera marina*	552	60.26697	8.33	Nucleus	-	198
ZmGRF10	Zosma970g00020.1	*Zostera marina*	495	53.93196	7.61	Nucleus	383	43; 240
ZmGRF2	Zosma2g02340.1	*Zostera marina*	329	37.03762	8.66	Nucleus	-	93; 216; 289
ZmGRF3	Zosma4g01130.1	*Zostera marina*	310	34.19019	8.18	Nucleus	207	133
ZmGRF4	Zosma8g00940.1	*Zostera marina*	482	53.09155	8.04	Nucleus	315	10; 212; 317
ZmGRF5	Zosma59g00170.1	*Zostera marina*	242	26.98431	8.81	Nucleus	-	104
ZmGRF6	Zosma67g00540.1	*Zostera marina*	360	40.60547	8.84	Nucleus	-	75; 84
ZmGRF7	Zosma81g00370.1	*Zostera marina*	280	31.90561	8.6	Nucleus	-	101; 176
ZmGRF8	Zosma120g00110.1	*Zostera marina*	347	37.60136	8.99	Nucleus	-	81; 90
ZmGRF9	Zosma328g00040.1	*Zostera marina*	320	35.68101	9.13	Nucleus	-	82

Table S2. Primers used for qRT-PCR

Name	Sequences
SpGRF1-F	CTACAAGTACCTCGACGCCAACG
SpGRF1-R	GGCTTCCACAGGCTTTCTTG
SpGRF2-F	GGTGCTTCCAAATGGGCTTCG
SpGRF2-R	CAGCGTAGGGATTCAGGAGA
SpGRF3-F	CCTTCCTCCCTGAACCTGA
SpGRF3-R	ACCCTGTTGGCGTCTGC
SpGRF4-F	GTTCACGGCGTCCCAATG
SpGRF4-R	GGCGTTTCCACAGGCTTTCT
SpGRF5-F	CCCATCACTCGCTTCCCTC
SpGRF5-R	CGGCGGGTTTGATGCTG
SpGRF6-F	CCTTTCACGGCGTCTCAAT
SpGRF6-R	GGGACGGAGATTTCCACAGG
SpACT-F	CCAGATCATGTTCGAGACCTTCAAC
SpACT-R	GGAGGGCGTATCCTTCGTAGATG
